# Identifying indigenous practices for cultivation of wild saprophytic mushrooms: responding to the need for sustainable utilization of natural resources

**DOI:** 10.1186/s13002-019-0342-z

**Published:** 2019-12-12

**Authors:** Deborah Wendiro, Alex Paul Wacoo, Graham Wise

**Affiliations:** 1 Centre for Industrial Microbiology and Bioprocess Engineering, Department of Product Development, Uganda Industrial Research Institute, P. O. Box 7086, Kampala, Uganda; 2 Department of Biochemistry, School of Biomedical Sciences, College of Health Sciences, Makerere University, P. O. 7062, Kampala, Uganda; 3 Department of Molecular Cell Physiology, Faculty of Science, Vrije Universiteit Amsterdam, Amsterdam, The Netherlands; 4 Universidad Regional Amazónica Ikiam, Klm 8 via Muyuna, 150150 Tena, Napo Ecuador; 5 Center for Global Health Equity, University of Wisconsin-Milwaukee, Milwaukee, WI 53211 USA

**Keywords:** Industry development, Wild saprophytic mushrooms, Edible mushrooms, Medicinal mushrooms, Traditional knowledge, Artisanal production, Natural resources management, Conservation, Sustainable development, Mushroom substrates

## Abstract

**Background:**

Due to increasing pressure on natural resources, subsistence agriculture communities in Uganda and Sub-Saharan Africa are experiencing increasingly restricted access to diminishing natural resources that are a critical requirement of their livelihoods. Previously, common-pool resources like forests and grasslands have been either gazetted for conservation or leased for agriculture, the latter in particular for large-scale sugarcane production. Satisfying the increasing consumer demand for grassland or forestry products like wild mushrooms as food or medicine, requires innovative ethno-biological and industry development strategies to improve production capacity, while easing the pressure on diminishing natural resources and averting ecosystems degradation.

**Methods:**

This case study addresses traditional knowledge systems for artisanal mycoculture to identify cultivation practices that enhance sustainable utilization of natural resources. Multi-scalar stakeholder engagement across government and community sectors identified artisanal mushroom producers across five districts in Uganda. Focus groups and semi-structured interviews characterized artisanal production methods and identified locally used substrates for cultivation of different mushroom species.

**Results:**

Artisanal practices were characterized for the cultivation of six wild saprophytic mushroom species including *Volvariella speciosa* (akasukusuku), *two Termitomyces* sp. (obunegyere and another locally unnamed species), *Agaricus* sp. (ensyabire) and *Agrocybe* sp. (emponzira), and one exotic *Pleurotus* sp. (oyster) that are used as food or medicine. The substrates used for each species differed according to the mushroom’s mode of decomposition, those being the following: tertiary decomposers such as those growing under rotting tree stumps or logs from forestry activity like the *Agrocybe* sp. known as emponzira which grows in forests, thickets, or near homesteads where big logs of hardwood have been left to rot. Also pieces of firewood are chipped off whenever need arises thus providing fuel; secondary decomposers growing on naturally composted grass associated with termites like the *Termitomyces* sp. known as obunegyere growing in protected sites in gardens, composted cattle manure for Agaricus sp. known as ensyabire in the kraal area where cattle manure is plenty, composted maize cobs for a locally unnamed *Agaricus* sp. on heaped cobs placed near homesteads; and primary decomposers growing on waste sorghum from brewing the traditional alcoholic drink, *muramba* for *Pleurotus* sp. (oyster), and banana and spear grass residue from banana juice processing like the *Volvariella speciosa* known as akasukusuku because it is associated with the banana plantation locally known in the Luganda language as *olusuku* and is usually heaped under ficus trees. Management practices also varied based on mode of decomposition and other ecological requirements such as the following: zero tillage and minimal disturbance in areas where obunegyere grow, heaping banana and spear grass residues under the cool ficus trees which also keep them away from banana stump that may cause infestation with nematodes and insects. Even within the generic practices accessibility by the users is critical for example placing logs near homes where children can use them to play, they can be used as fire wood and to even get off-season mushroom as household waste water can make the mushrooms grow.

**Conclusions:**

Our description of artisanal mycoculture methods that respond to conservation and utilization pressures, demonstrates the value of addressing traditional knowledge to improve ethno-biology and mycoculture industry practice. Traditional communities engage in multiple technological and organizational innovations and practices for sustainability and in the case of mushroom production to conserve the environment and culture, ensure variety, food and nutrition security, and income. The results of this study present opportunities to preserve ecosystem quality while developing an artisanal mycoculture system. They have also identified aspects of artisanal mycoculture that most urgently require further ethno-biological study and industry development. Future research and industry development can utilize the result of this study to boost artisanal production of wild saprophytic mushrooms in Sub-Saharan countries, for food or medicinal consumption, and environment conservation. Further development of production efficiencies in context with sustainable natural resource management is recommended.

## Background

In 2015, the world awoke to a universal call to action coined as the Sustainable Development Goals (SDGs), an extension of the prior Millennium Development Goals (MDGs). The objective of the SDGs is to end poverty, protect the planet, and ensure that all people enjoy peace and prosperity. Key goals include Climate Action, Reduced Inequality, Industry Innovation, Responsible Production and Consumption, and Peace and Justice [26]. Worldwide, much funding and other resources have been invested in projects and programs aimed towards achievement of MDGs and SDGs. Successes have been reported [76], yet many countries, for example Nigeria [66] and Uganda [8], have reported failed or lukewarm progress. With increasing recognition that present extractive and consumptive development paths around the world are not sustainable, and the urgent need to address issues of sustainability, subsequent calls for an integrating framework [10, 63] even by the European Union [22, 23] have been sounded, triggering a search for appropriate developmental models. These developmental aims are reflected in increasing research on sustainable innovation systems, research to identify causes of failure [81], and research to identify determinants of success [32, 49].

Indigenous innovations have persisted in very low–income countries in Africa like Uganda and Tanzania [45], and in Latin America [21], despite a blitz of undermining national policies. Examples of such policies in Africa are the structural adjustment policies [16], and the National Economic Survival Plan of Tanzania [45], which have reportedly intensified poverty, disease, maternal and infant mortality levels, and deforestation. Repressive laws to ensure compliance with such policies have also historically stifled innovation [77]. In Latin America, the rich agrobiodiversity associated with traditional farming systems has been greatly undermined by national agricultural policies that promote large-scale monoculture farming [82]. Yet examples of positive benefits from traditional knowledge are many. In Niger for example, it was realized that community members, their ideas, knowledge, experience, and enthusiasm were the greatest resource available to address land degradation while alleviating poverty [83]. In his documentary, “Thirty Years of Banana,” local Ugandan artist Alex Mukulu showed that local people’s livelihoods and resilience were enhanced by subsistence economic production that was based on artisanal innovations and informal economies [56], as also described by Lemarchand [43]. This has triggered social, economic, and political transformation [55] and ushered indigenous Ugandans into the regional market in the famed “Magendo Economy” [5, 6, 36]. Great innovations have also been historically derived from artisanal guild community contexts in developed economies like Germany, Britain, and France [20, 38, 39]. It is our responsibility to explore ethno-biological practices and eco-industry development interventions in Uganda that also draw on artisanal knowledge, to generate future ecosystem-business innovation that is contextually appropriate at both local and global scales.

This article contributes to an analysis of intervention options, and to a formulation and stimulation of environmentally sustainable and economically feasible mechanisms for wild saprophytic mushroom cultivation. We highlight the importance of recognizing existing traditional knowledge systems and building on them. We argue that organically rooted development processes that are grounded in local economic, environmental, cultural, and technical contexts present greater opportunities for socially inclusive and sustainable development. Indigenous innovations in agroecology hold great value not only for the development of agricultural biodiversity but also for environment conservation, community economic resilience, population health, and socioeconomic transformation as foundations and purveyors of change. This is because indigenous innovations comprise an elaborate web of varieties and types of innovation, as described by Popadiuk and Choo [74], which functions as an innovation ecosystem for natural resource management. Interest in traditional innovations derives from their existing ecological, socioeconomic, and cultural value, and the fact that these innovations have been tested over millennia through local community participation [19]. Therefore, they represent solutions that have a greater likelihood of strong uptake in the communities that need them. However, traditional innovations for wild mushroom cultivation in Uganda have not been identified, described, and documented. Such analysis is necessary to facilitate intervention options and to formulate effective industry development pathways.

In this article we identify and describe the artisanal cultivation of wild saprophytic mushroom species as examples of innovative artisanal farming systems in Uganda. We note that access to natural resources to forage for mushrooms is increasingly being curtailed by natural resource management policies, conservation regulations, and agricultural development programs that are not in accord with ecosystem health, traditional systems of property ownership, or natural resource utilization and conservation. These factors undermine the viability of traditional methods for wild saprophytic mushroom cultivation. It is shown that as traditional farming communities are assimilated into the wider global economy, their farming systems for wild saprophytic mushroom are simply not sufficient to ensure local food security and community economic resilience. It is necessary to identify and develop versatile practices which adapt traditional knowledge to address modern supply-side natural resource constraints and demand-side production volume pressures. This study identifies artisanal methods for the cultivation of wild saprophytic mushrooms, which are based on indigenous knowledge used in traditional farming systems. It considers how to augment them using a modern understanding of higher yield mushroom mycoculture, while remaining considerate of the availability of local resources as environmental and agricultural inputs.

### The role of mushrooms in ecosystems health and stability

Mushrooms are a vital component of biologically diverse and healthy ecosystems due to their role in the cycling of carbon, nitrogen [27, 30, 41, 42, 61], and other elements, globally and specifically in Sub-Saharan African regions like the Lake Victoria Basin of Uganda [3, 18, 34, 78]. Their cultivation also represents a valuable contribution to agricultural biodiversity, supporting local food security and community economic resilience [33]. The tradition of wild edible mushroom consumption as foods and medicines has existed for centuries, across many countries (see Martins [52] for a review). In modern economies, the Food and Agriculture Organization documented 83 countries worldwide where wild mushrooms are consumed and provide earnings to rural people, also listing 30 countries in Sub-Saharan Africa including Uganda [9]. Within Uganda, foraging and gathering of at least 10 mushroom species supports the livelihoods of many local people [37, 65, 68]. Many species of mushrooms have great economic and cultural importance providing food and medicine to many communities [18, 65, 68]. Mushrooms maintain ecosystems balance and stability, being primary, secondary, and tertiary saprophytes (growing on dead biomaterials) and mycorrhizae (forming mutually beneficial associations with other plants). Depending on the species, mushrooms are natural resources that recycle carbon, nitrogen [40], phosphorus and other nutrients [30, 60, 78].

Many strategies have been applied to manage natural resources internationally, using community participatory approaches that consider stakeholder values, model local needs, and use platforms for participatory decision making [1, 2, 4, 25, 35, 84]. Studies have also been undertaken in Sub-Saharan African countries that address participatory approaches to natural resource management including the voices of indigenous communities [12, 13, 48, 73, 80, 83]. These studies have emphasized the enhanced use of traditional knowledge in conservation of natural resources and sustainable harvesting [70]. While Pinton [71] has eloquently articulated the many political, institutional, and economic detractors that undermine the use of indigenous knowledge and traditional farming systems, importantly he highlights the need to rehabilitate traditional knowledge in context with modern political and economic forces. Madulu [48] has further illustrated within Sub-Saharan Africa that population pressures and land use conflicts between conservation and economic development must be resolved through community integrated land use planning and management, considering traditional knowledge and lifestyles.

Yet due to changes in environment and land management practices, there is a strong need for artisanal agricultural practices to reconceptualize traditional and modern knowledge so that it can succeed against modern market forces, to reverse current trends of agrobiodiversity loss [75], diminishing community economic resilience and loss of local food security [33, 82], and environmental degradation [48]. Novel agricultural practices must embrace practices such as intercropping and agroforestry as widely practiced in Africa, Central and South America [7, 14, 51, 79, 82], as well as supporting sustainable biocommerce [47, 69, 86].

### Situation analysis, historical perspectives, and emerging trends

There is a wide diversity of mushroom species in Uganda [18, 37]. In the Teso region of Eastern Uganda for example, 28 species have been identified, of which 22 are edible, 12 have medicinal value, and two are currently commercially exploited [68]. An ecological study has documented 10 edible species across five genera, noting species diversity in grasslands, which is significant for socioeconomic reasons [18]. In Uganda all mushroom species apart from the commercial oyster species are collected from the wild [62] and their availability is seasonal [68]. Thus, local populations depend on such wild resources for their food and medicine, and to supplement their income [57, 58, 65, 68]. Currently commercial varieties of mushrooms are similar to wild types also sold in Uganda. They are imported from Kenya, South Africa [64], and the Netherlands (personal observation), and mainly sold in urban areas by the dominant supermarket chain.

Prospects for mushroom gathering from the wild are fading due to diminishing land access [64], and changing ecosystems [65, 68]. As natural resources for mushroom foraging diminish, foraging pressure on remaining habitat increases. Furthermore, foraging is increasingly prohibited in remaining areas, which have been gazetted as forests to be managed and protected by the state or by affluent people in local communities. This increasingly restrictive foraging environment is detrimentally affecting sustenance and livelihoods. Apart from oyster mushrooms, other varieties of mushrooms have not been widely cultivated or domesticated due to lack of technical skills, planting materials, capital, and the poor availability of wild mushrooms [53, 68], although many people, including those interviewed in this study, have expressed the need to grow them. It has been observed that local varieties are preferred by consumers because they have a familiar flavor when eaten as a whole food or as a condiment, or because they have locally known medicinal value [65]. Important for the development of community economic resilience, local varieties of wild saprophytic mushrooms earn more income for producers than commercial oyster mushrooms. The strong consumer market for local wild varieties of mushrooms is evident in the thriving sales of wild mushrooms along motorways (Nshemereirwe, 2004), and in urban markets like Nakasero and Owino (Saint Balikuddembe) markets in Kampala. This situation analysis of mushroom production highlights a great demand for wild saprophytic mushrooms that is not being satisfied at the current level of production.

This paper identifies and characterizes artisanal practices for the cultivation of wild saprophytic mushroom species to ensure conservation, enhance their role in ecosystems, and promote a balance between livelihood enhancement and environment conservation. This study indicates the need to reach beyond botanical (mycological) and conservation approaches, to integrate the growing of currently wild mushrooms into subsistence agriculture, agro-forestry, urban agriculture, and other land management practice, as a development strategy for food and nutrition security, health, and poverty reduction.

## Methods

### Conceptual framework for analysis

The scientific discourse on agriculture for development conceptualizes the advancement of new agricultural processes as being a dynamic and relational activity that is based on local participation, depending upon the context and communities involved, rather than a one-way information flow informed by Western methodologies [5, 6, 20, 36, 38, 39, 43, 55, 56, 74]. This study is grounded by a theoretical understanding of tacit and explicit knowledge transfer [59, 74]. We conceptualize knowledge transfer within an innovation systems conceptual framework [46, 50, 72], which places emphasis on knowledge generation, dissemination, uptake, and diffusion. In doing so, this study uses a process of participative engagement with innovation system actors to both identify mushroom cultivation as a focus for this study, and to identify innovators and innovation practices.

### Innovation system context

This study is an analysis of artisanal mycocultural innovation by traditional knowledge holders. We used qualitative methodologies as described by Hesse-Biber [29], and separately by [28], involving semi-structured interviews with individual participants or key informants, and focus groups. All data was collected in the Ugandan frontier districts of Kabale and Arua, and in the inland districts of Kamuli, Buikwe, and Mubende. The first three districts are characteristic due to their geographical proximity to regional markets. Buikwe and Mubende are characteristic because of the known existence of producers of wild saprophytic mushrooms. Kamuli district is also characteristic for several other reasons: it is a high population density and high poverty district [10, 15, 17, 54], with high socioeconomic and environmental vulnerability as described in a participatory rural appraisal by Lentz [44]. It also has emerging issues resulting from sugarcane production that is replacing other agronomic systems, causing socioeconomic and political upheaval.

Uganda is a small land-locked country located at the heart of East Africa. It has varied climate ranging from humid tropical in its equatorial region, semi-arid towards the north, and temperate at high altitude in the western mountainous Rwenzori, Muhabura, and Elgon areas. The terrain of Uganda can be considered a plateau, rimmed by mountains, creating a variety of microclimates that have contributed to the generation of many different climate-sensitive agricultural innovation systems. This is why identification of mushroom cultivation systems was addressed across multiple districts of Uganda.

The presidential and parliamentary democracy of Uganda regionally diffuses responsibility for the governance of functions such as local economic development. At a district level, governance is headed by a Resident District Commissioner (RDC). Within each district we presented planned field activities to the RDC to ensure the security and scientific independence of our activities. Interviews were held at a district level with the Chairperson of the Local Council Five. Being knowledgeable about the cultural and socioeconomic context of the district, they were able to identify innovation hotspots by sub-county, or prominent innovators and key production activities. Interviews were then held at a sub-county level with the Chairperson of the Local Council Three, who also identified innovation hubs and the names of household heads or innovators and their locations. Then, interviews were held at a village level with the Chairperson of the Local Council One of the identified villages, who also identified the locations of innovators. Other interviews were held with the District Chief Administrative Officer, the District Production Officer, and other government officers as referred by the responsible government authorities for agriculture and agro-industry development. The participative, innovation system–sensitive, and community-integrated methodology described above was used to identify innovative artisanal mycoculture practices and select participants for this study based on knowledge that is held across many complex government, cultural, language, and social divisions. Such an approach is favorable to randomized selection of participants in such a complex cultural, political, and socioeconomic context, where the identity and location of innovators as prospective participants is unclear.

### Innovation identification and participant selection

Eight focus groups having between 8 and 20 participants were held across all five districts, at village locations that were identified by government innovation system actors. Through village-level focus groups, information could be accessed from informal innovation system actors such as clan members and *nigiina* groups (community collectives), which play an important role at a village level in information flow, user rights and benefits, and innovation processes. In accordance with Gill et al. [24], focus groups were used to generate information on collective views, group norms, and community narratives on common innovation challenges, and to identify innovation practices used in the local region. Focus groups yielded valuable information on innovations that are considered public knowledge or not easily copied; in such cases group participation was strong since participants wanted to learn from each other. In the Lusoga dialect this business knowledge sharing is described as “*omughesi azimba kungira kulagirirwa* [an artisan establishes a shop by the roadside to be guided].”

### Semi-structured interviews

In many focus groups, respondents were uncomfortable with the presence of potential “competitors” when discussing innovations that are not normally discussed publically. In such cases, focus group participants would remain silent. For this reason, individual semi-structured interviews with key informants were important, in particular for discussions on mushroom growing practices and the preparation of herbal medicines as follows:

#### Demographics and gender dimensions

Within the existing innovation systems framework, it was found out that females are located at the fringes of all production processes. All government and local government officers interviewed were male; in Arua the two respondents were male herbalists; in Kabale and Kamuli of the two respondents each, one respondent was male and another female respectively, and all were progressive farmers. In Buikwe and Mubende all three respondents were female agro-processors and progressive farmers.

This study undertook eight semi-structured interviews with key informants. The objective was to collect firsthand information from practitioners to identify practices for sustainable cultivation of wild saprophytic mushrooms and describe traditional knowledge systems that could be commercially developed and replicated. Especially relevant dimensions for the semi-structured interviews were as follows: capabilities in knowledge creation, knowledge about the market, knowledge about the reasons for certain actions, identification of natural resources used and how they are used, seasonal variations, and who does what and why? Other areas covered were attitudes, organizational dynamics, product and process-based attributes, market characteristics, and marketing strategies.

#### How taxa were identified

For identified taxa information collected was collated with existing literature about characteristics of the organisms; we also consulted mycologists specifically Dr. Nakalembe of Makerere University who knew the specific name of akasukusuku; the pictures of some of them were also compared with those of identified species.

### Data collection

All data from participants was simultaneously translated from local languages into English and recorded in writing with as much detail as possible. Audio transcripts were not used because of strong reservations that many community participants had regarding audio recording. Instead, detailed note-taking facilitated the transparent communication of greater detail about innovations. The information was manually coded and organized into conceptual categories and structured and grouped into thematic areas for reporting.

## Results

### Production system selection

Fifteen potentially innovative biocommerce production systems were identified as a result of interviews with innovation system actors and focus groups. Production systems included the following: bark cloth making and dyeing, textile fabrication, preparation of herbal medicines, mushroom cultivation for medicines and for consumption as food. Mushroom cultivation was most commonly identified in focus groups, reinforcing its value as a focus for this study, and as a target for future subsistence agriculture intensification, agroecology conservation, and agro-industry development activity. Specifically, edible or medicinal wild saprophytic mushroom production met minimum social, environmental, and economic selection criteria that considered support for gender equity, nutritional value, economic benefit to smallholder producers, environment impact of by-products, existing market demand, future market growth potential, sustainable consumption of natural resources, potential for value-added product development, available technological capability, upstream supply chain access, and downstream value chain availability. Wild saprophytic mushroom production met all of these selection criteria as an income-generating artisanal agronomic practice, rooted in traditional knowledge, considerate of gender equity and modern economic pressures, contributing to recycling of waste, and enhancing livelihoods and environmental sustainability.

### Mushroom production systems

This study has identified six production methods for saprophytic mushrooms, based on the use of different substrates for mushroom production. Ordinarily generated as by-products of existing local community subsistence production or agroindustrial activity, these substrates represent low-cost, locally accessible, and environmentally sustainable mechanisms to further develop artisanal mycoculture processes that can transition communities from wild harvesting methods. The six substrates for mushroom cultivation are as follows:

- Naturally composted grass or forest litter occurring in association with termites
- Composted cattle manure as a byproduct of traditional cattle keeping and beef or dairy production
- Banana juice residue and spear grass as a byproduct of *tonto* processing
- Waste sorghum as a byproduct of *muramba* production
- Composted maize cobs as a byproduct of maize milling operations
- Deadwood from hard wood trees as a byproduct of agro-forestry operations or small holder property maintenance

Collectively, these substrates were used by participants of this study to cultivate five types of wild saprophytic mushrooms, in addition to commercial oyster mushrooms, as described in Table 1.

**Table 1:** Description of mushroom substrates, target species, scientific and common names

Substrate	Scientific name	Local Name	Local language
Grass waste or forest litter	*Termitomyces* sp.	*obunegyere* *obwisonkere* *obubaala*	Lukiga Lusoga Luganda
Cattle manure	*Agaricus* sp.	*ensyabire* *entyabire*	Lukiga Lusoga
Banana residue and spear grass	*Volvariella speciosa*	*akasukusuku*	Luganda
Sorghum waste	*Pleurotus* sp.	Oyster	English
Maize waste	*Agaricus* sp.	Unnamed	N/A
Deadwood from hardwood trees	*Agrocybe* sp.	*emponzira*	Lusoga

### Grass waste or forest-litter mushroom production

*Obunegyere* are *Termitomyces* mushrooms species which are secondary decomposers grow widely in all of the districts investigated since these mushrooms tolerate a wide variety of climatic conditions. Historically, these mushrooms have been foraged and gathered from forests and grasslands. It was reported by participants that people modify the microenvironment of production sites within grasslands or forests to enhance *obunegyere* mushroom productivity. *Obunegyere* mushrooms bloom during the rainy season, and during this reproductive growth phase, growers lightly place piles of grass or banana leaves over sites where pinheads are seen; then, they water them to control moisture content until they grow to an appropriate size and are harvested. The Basoga refer to emerging mushroom pinheads as *omuswiga*; however, in Kabale District growers refer to signs left behind by “ants” (termites or emishwa) to determine when to apply environmental control to enhance the reproductive phase of growth.

According to local beliefs regarding mushroom cultivation using this method, there is an association between the waste grass or forest litter, termites and mushroom development, which is often referred to by the phrase *Emishwa nekoora ebituzi* (termites produce mushrooms) by the Bakiga. According to traditional knowledge, grass waste or forest litter is eaten by termites, stimulating the growth of the termite colony with which mushroom growth is associated. Elders enthusiastically protect from human interference, sites known to have termite activity, leaving termites to establish a colony and ensuring that year after year they can harvest mushrooms from these sites. The Bakiga in Kabale believe that the chamber of a termite colony is “clean” with very many holes and tunnels, and therefore, termites are responsible for the growth of small *obunegyere* mushrooms. *Obunegyere* mushrooms will grow in the same location, at about the same time of year, perennially if there is no interference from incompatible land use like tillage. Hence, experienced people purposively inspect cultivation sites at a time of year that is most associated with mushroom growth.

It was locally reported that despite the use of this method to manipulate the microclimate of sites within forests, production of wild mushrooms has reduced due to conversion of forests and grassland for agriculture, and deforestation for charcoal burning. Officials of the Ugandan National Agricultural Advisory Services supported the conclusions of local growers, believing that it is not climate change that is causing low mushroom production but diminishing land access and availability, due to population pressures. They also specifically highlighted the negative impact of agricultural pesticide use, stating that “the termites have been destroyed with insecticide which may reduce mushroom growth in the wild.” This finding is corroborated by the findings of [31, 67, 85] who have illustrated that termites have a mutualistic or symbiotic association with fungi. Furthermore, local growers reported that the environmental control method cannot overcome the seasonality of *obunegyere* mushrooms; therefore, current production cannot satisfy local needs for sustenance, and a growing market.

### Cattle manure mushroom production

A mushroom cultivation technique using cattle manure was described in Kabale district. Internationally, cattle manure is commonly recommended as an additive to substrates for commercial mushroom cultivation, especially in *Agaricus bisporus* production systems [11]. *Agaricus* sp. such as common button mushrooms are secondary decomposers growing on composted biomaterials. Substrates that are entirely composed of cattle manure are uncommon, presumably due the limited availability of cattle manure and the low cost of straw substrate in most Western commercial growing environments. However, in Kabale District, local growers reported that cattle manure is an easily accessible and low-cost local substrate for mushroom cultivation. Study participants reported that cattle manure is simply placed in a pile and left to compost spontaneously. After the composting period, the mushrooms locally known as *ensyabire* (*Agaricus* sp.) were reported to grow on the waste by natural spawning. Compost heaps are regularly inspected for fruiting *ensyabire* mushrooms. Typically, the reproductive period of these mushrooms occurs during the rainy season when the temperatures are low, in the range of 17 to 20 °C, and when there is high humidity for at least 4 consecutive days. Growers were unable to identify the origin of the *ensyabire* mushroom spawn, leaving them unable to control the inoculation of their substrate by their target species.

It was also reported that in some areas, *ensyabire* mushroom cultivation is achieved by composting grasses and cattle manure together for a long period of time during the wet season. However, specific ratios of substrate constituents and composting time were not stated explicitly. Thus, it appears that 100% manure may not be required for *ensyabire* mushroom production, although the minimum manure composition to maintain optimal yield is unquantified.

### Banana residue mushroom production

**Fig. 1:**
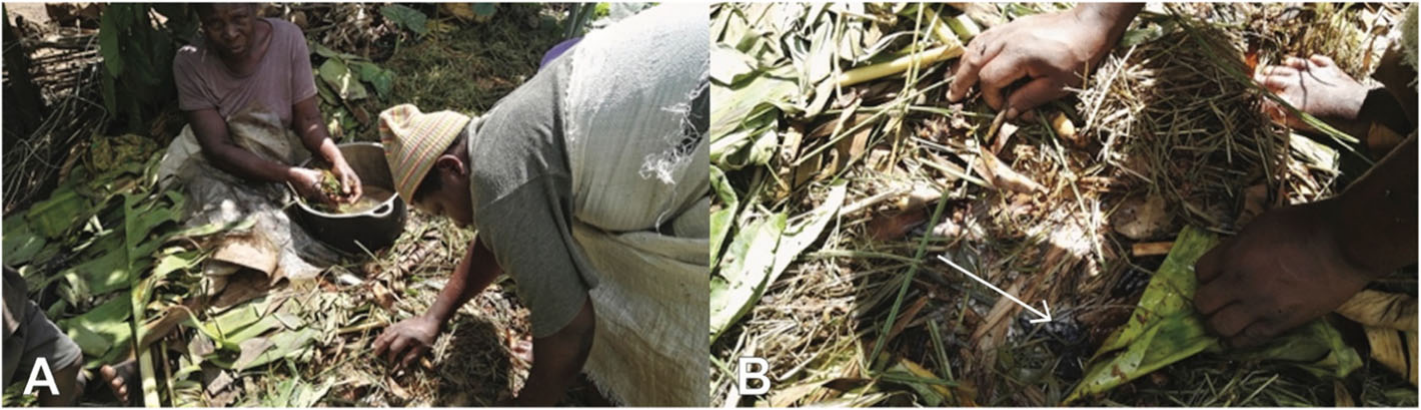
**a** Banana juicing for the processing of *tonto* fermented beverages in Buikwe district of Uganda. **b** The arrow indicates the presence of the mycelium of *akasukusuku* in discarded spear grass and banana residue

A mushroom cultivation technique was described in Buikwe District using banana residue from the *kisubi* banana cultivar, known locally as *embidde*, used in the processing of *tonto* fermented beverages. Buikwe is a banana growing region where banana residue is easily accessible at low cost due to its generation as a processing byproduct of this common local beverage. *Embidde* are juiced using spear grass which is abrasive enough to express juice from the bananas when they are wrung and squeezed (locally described as okusogola in Luganda and okukunha in Lusoga) by hand, until juice oozes out a process called *okummuka* in Luganda and *okutumbuka* in Lusoga (Fig. 1). However, in larger-scale operations a boat-like vessel (*eryaato*) is constructed out of wood. On occasions the banana fruit is crushed with the male banana flower to avoid precipitation and coagulation of the juice. In Lusoga this is called *okugwa eitete*. In the Busoga region there is alternative use of an herb that is locally known as *ensasira*, the bark of which is used to avoid precipitation and coagulation. There is opportunity for local people to conserve this particular herbal species. The spent grass mixed with banana residue is then heaped in a cool place under trees (usually *Ficus* sp.) which are leafy and provide a cool environment within the banana plantation and left to spontaneously compost. Heaping it under the tree also compartmentalizes the waste away from the banana stump to prevent infestation with nematodes and insects, and prevents banana roots from growing on surface which may lead to premature falling down, loss of the crop, and destruction of whole plantation. The primary decomposer, *akasukusuku* (*Volvariella speciosa*) species of saprophytic mushroom grows on the waste by natural seeding. The local people who purposively heap the waste continue to check them for fruiting bodies. The mushrooms do not grow during the dry season due to high temperatures and low humidity. They grow during the rainy season when the temperatures are low, in the range of 17–20 °C and with high humidity for at least 4 consecutive days. Under these conditions, mushrooms bloom and can be harvested.

### Muramba sorghum waste mushroom production

**Fig. 2:**
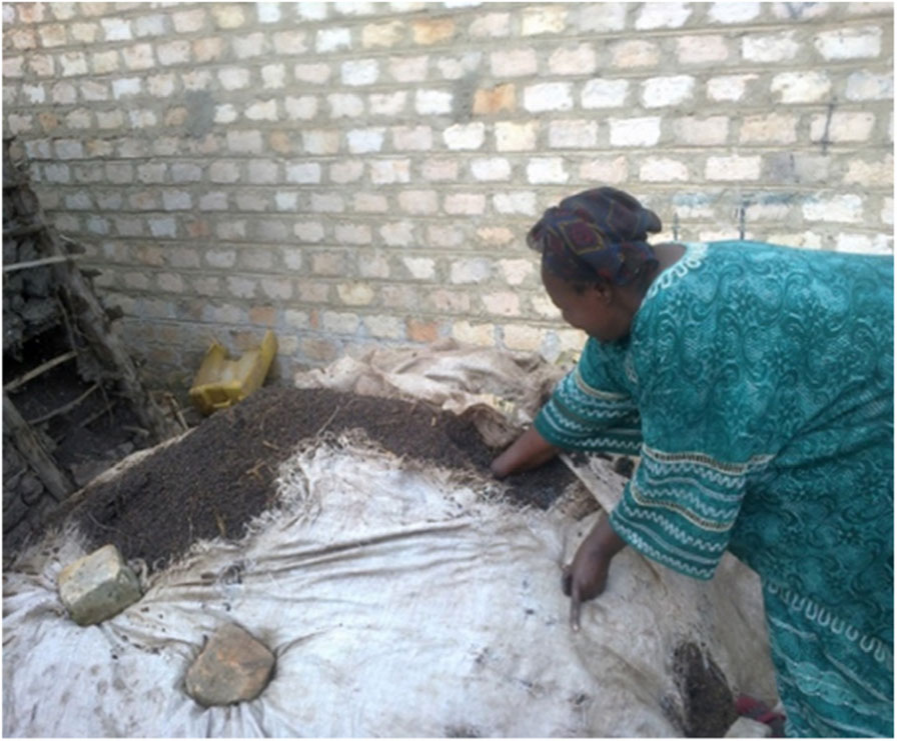
An oyster mushroom producer in Kabale District composts *muramba* sorghum waste from the production of *muramba* beverages. The composted substrate is used for oyster mushroom production

During the 1990s, through the Ugandan Ministry of Agriculture, Animal Industry and Fisheries, there were developmental programs to introduce oyster mushroom (*Pleurotus* sp.) production as an income-generating activity among low-income and resource-poor communities. These programs targeted women as growers to increase household incomes. They were trained to use agricultural waste for producing oyster mushrooms. One such system that continues to be employed uses waste from sorghum seeds used to make the fermented beverage called *muramba*. The sorghum waste is heaped and composted and then mixed with other agricultural waste such as maize stalks and tied in polythene bags called “gardens” and hung on posts in a shed. Community-based microenterprises use basic grass thatched constructions to provide shade. On a larger scale, houses or other modern structures are used as shown in Fig. 2.

### Maize waste mushroom production

**Fig. 3:**
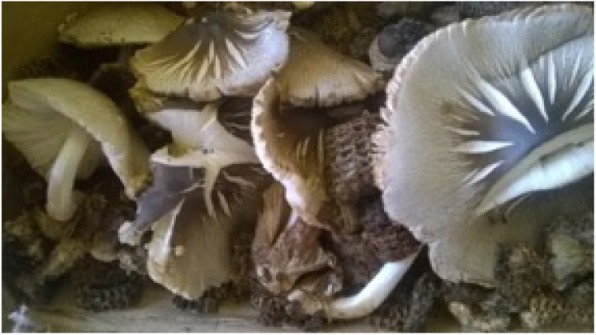
*Termitomyces* sp. mushrooms growing spontaneously on waste maize cobs

In the Mubende District of Uganda, a female local grower described a simple method for growing *Agaricus* sp. mushrooms on naturally composted maize cobs. A local maize miller generates a large quantity of maize cobs as a by-product of the milling process. The waste maize cobs are intentionally piled under a tree nearby after milling, for reuse as a substrate in mushroom production. During the rainy season, *Termitomyces* sp. mushrooms spontaneously sprout on the cobs (Fig. 3), which are harvested by local people for consumption or commercial sale.

### Deadwood mushroom production

**Fig. 4:**
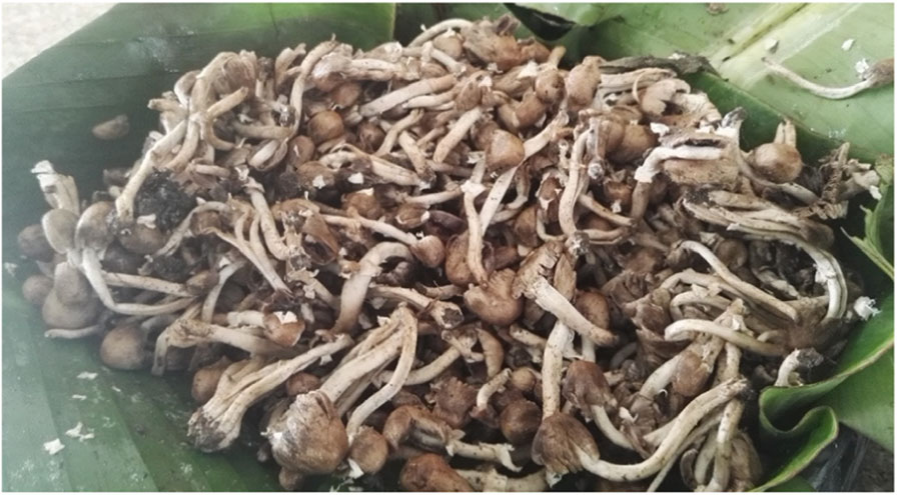
*Emponzira* mushrooms that grow on deadwood from hardwood trees

A mushroom cultivation technique was described in Kamuli District whereby hardwood trees that have either died off or have been felled during clearing of woodlands or forests for agricultural purposes are used as a substrate for mushroom production. A small brown mushroom variety locally known as *emponzira* (Fig. 4), which is tentatively identified as *Agrocybe cylindrical*, grows on this deadwood, and they are collected mainly by women who look for them when they go to collect firewood or other products like wild yams and fruits. Recently due to increasing pressure on land, trees are cut to clear for agricultural activities. Some logs are kept near homes and used for firewood. After a period of time emponzira which are tertiary decomposers can grow on the logs. Knowledgeable people place agricultural waste and deadwood under other trees left uncut thus creating a cool environment for *emponzira* to grow. The mushroom requires a cool environment that is undisturbed.

## Role of the traditional techniques and products in local people’s subsistence

The role of traditional techniques and products in local people’s subsistence is elaborate and diffuses through the cultural, social, and economic aspects of their very existence. They engage multiple technological and organizational innovations and practices for sustainability and in the case of mushroom production to conserve the environment and culture, ensure variety, food and nutrition security, and income.

In the grass waste or forest litter mushroom production systems zero tillage or minimum interference ensures access to resources such as rare mushroom species, medicinal herbs, fire wood, income, and conservation of endangered species such as termites and intricate biomes. It is reported that termites “fetch” water from underground or great distances during dry weather thus keeping microenvironments green, in that way providing foliage to feed browsing and grazing animals even during prolonged drought.

In the cattle manure mushroom production system, productivity is increased by harvesting mushrooms from waste.

In the banana residue mushroom production system enhanced composting increases recycling of nutrients such as nitrogen and microelements, conservation of moisture by providing mulch, and control of pests and diseases. The banana plantations last longer with reduced labor inputs and higher yields.

### Summary of results

The knowledge on mushroom production systems using different substrates that was gathered from interviews and focus groups has been analyzed in context with environmental and agricultural inputs for mushroom production. Table 2 demonstrates the level of control imparted by all six production systems, considering each environmental or agricultural input. Control of the nutrition of the substrate was strong for all production methods and mushroom species, perhaps presenting a strong basis from which to plan future industry development activities. However, spawning methods were not controlled for any of the wild mushroom species. Spawning method was only controlled for cultivated oyster mushrooms following a government-sponsored industry development program focused specifically on the commercial development of this species. Environmental inputs such as light, moisture, temperature, and humidity were partially controlled or not controlled depending on the effectiveness of available natural control factors such as shade provided by covering trees or humidity maintained by grass that is heaped on top of some production sites.

**Table 2:** Degree of control imparted by each of the mushroom production methods

Substrate and mushroom type	Nutrition of substrate	Control of contamination	Temperature	Light	Moisture content	Substrate pH	Substrate air-gas exchange	Humidity	Spawning method
Grass waste (*obunegyere*/*obwisonkere*)	C	C	PC	PC	PC	PC	PC	PC	NC
Cattle manure (*ensyabire/entyabire*)	C	PC	PC	NC	NC	PC	PC	NC	NC
Banana residue (*akasukusuku*)	C	NC	PC	PC	PC	PC	PC	PC	NC
*Muramba* sorghum waste (oyster)	C	PC	PC	C	C	PC	PC	PC	C
Maize waste (*local name not yet identified*)	C	NC	PC	PC	NC	PC	PC	PC	NC
Deadwood of hardwood trees (*emponzira*)	C	NC	PC	PC	PC	NC	PC	PC	NC

*C*, controlled; *PC*, partially controlled; *NC*, not controlled

In Table 3, each production system and mushroom species has been summarized to highlight the practices observed by this study in context with reported commercial uses for the five studied wild mushrooms, ecosystem relevance, and relevance for gender equity.

**Table 3:** Framework linking ecosystems to services and agro-processing goods and their production chain

Grass waste production of *obunegyere* mushrooms (associated with termites)	
Observed practice: Grass is heaped near anthills where *obunegyere* are known to grow; no tillage or pesticide use nearby to preserve the site; pinheads are irrigated when they appear during dry weather.	
Commercial use: Emerging value-added soups and mushroom flour products; placement of branded products in supermarkets.	
Ecosystem relevance: Termites are erroneously believed to produce the mushrooms; locations containing termites have assumed spiritual status; recycling of grassland biomass aids mushroom production.	
Gender relevance: Women look after production sites and collect mushrooms; wooded production sites also produce timber for firewood which benefits women; men also collect timber for construction.	
Cattle manure production of *ensyabire/entyabire* mushrooms	
Observed practice: Manure from cattle is heaped; no burning of cattle manure as is usually the case; manure is sometimes mixed with grass; heat from compositing partially controls contamination; no tillage near production sites created by heaped manure.	
Commercial use: They are sold fresh or made into flour and mushroom soup in rare cases. After mushroom production, manure-based waste mushroom substrate is used as a fertilizer for banana or coffee production.	
Ecosystem relevance: Cattle keeping, mushroom production and agriculture of banana or coffee become integrated production systems reducing demand on ecosystem services.	
Gender relevance: Cattle usually owned by men; women associated with cleaning activities and therefore collection of manure.	
Banana residue production of *akasukusuku* mushrooms	
Observed practice: Banana residue is heaped with grass under leafy trees; Shade from trees partially controls light, temperature and humidity; No tillage or burning at production sites; Irrigation is provided as necessary.	
Commercial use: They are sold fresh or sun dried in urban markets. *Embidde* bananas that are used in *akasukusuku* production are processed into sun dried chips (Obukeke), eaten raw or boiled and mashed; banana peels are also used to feed small ruminants and poultry; banana juice is used to make *tonto* a local alcoholic beverage.	
Ecosystem relevance: *Embidde* bananas are necessary for mushroom production; there is belief that this variety maintains garden health; the Basoga people believe the plant is male; they use spear grass as a tool for juicing which breaks down the grass with fruit sugar for better decomposition; the banana agricultural system uses the *Ficus spp.* trees for protection as wind breakers and feed for small ruminants; recycling of banana agriculture biomass aids mushroom production. Compartmentalization of waste helps control pests and diseases and heaping enhances composting for quick recycling	
Gender relevance: Women own banana gardens; *Ficus* trees are usually owned by men who decide how to use them, including the production and sale of bark cloth; Women use the leaves to feed small ruminants they own; Since the bark has lost economic value, few people value bark cloth; Trees are cut for charcoal by men often without consulting women.	
*Muramba* sorghum waste production of oyster mushrooms	
Observed practice: Waste sorghum is roasted, grinded, mashed and fermented together with malt to produce a *muramba* compost substrate. Composting *muramba* waste is used to grow oyster mushrooms; heat from compositing partially controls for contamination.	
Commercial use: Oyster mushrooms are produced in larger commercial volume and sold in supermarkets; *Muramba* is a fermented beverage consumed by all age groups and even used as a weaning food; there is emerging commercial development of *muramba* beverages for sale in supermarkets.	
Ecosystem relevance: The sorghum requires a lot of soil nutrients; compositing and recycling waste sorghum benefits overall use of soil nutrients; sorghum production often involves clearing forest cover; recycling of sorghum biomass aids mushroom production.	
Gender relevance: Cultural gender bias results in *muramba* production by women; men are the main consumers of fermented *muramba* beverages; children also drink muramba with low alcohol concentration.	
Maize waste production of *Agaricus* spp. mushrooms	
Observed practice: Maize cobs from milling processes are heaped as a substrate for mushroom production.	
Commercial use: They are sold fresh and eaten locally. Exhausted maize-based substrate is used as fertilizer.	
Ecosystem relevance: Maize waste is considered be a hazard because in areas where it is generated away from the garden it attracts vermin like snakes, rats, and litters the home microenvironment; reuse as a substrate for mushroom production reduces an environmental hazard and creates economic income.	
Gender relevance: In most agroindustrial processes such as maize production, women occupy support roles such as cleaners, peelers, or packers; Therefore, women have close contact with maize waste and harvest the mushrooms. Ponce et al. [73] also made similar observation.	
Wood stump (decaying wood) production of *emponzira*	
Observed practice: Gathered naturally from decaying wood in grasslands and forests or from intentionally cut and covered wood stumps.	
Commercial use: In other countries such as China, similar mushrooms are grown on commercial scale and sold fresh or dehydrated; the local people here indicated that sale is very minimal and localized because they are rare.	
Ecosystem relevance: Fungal decomposition of deadwood in forests has been now purposefully enhanced to improve commercial yield; timber that has been purposed for firewood but is not being immediately combusted can be given a dual use as source of mushroom production; dual use of forestry product improves economic viability and sustainability.	
Gender relevance: Men fell timber. Women have support roles in removing grass waste, weeding and preparing wooden stumps for mushroom spawn.	

Finally, an appraisal of the role of traditional techniques and products in local peoples’ subsistence was done by assessing them against existing knowledge, leading to exposition of their value for subsistence agriculture intensification, agroecology conservation, and agro-industry development activity.

## Discussion

### Controlling environmental and agricultural inputs

The agricultural transition from opportunistic foraging for mushrooms to commercial mycoculture production systems, can be described in terms of an increasing degree of control over agricultural and environmental inputs. Opportunistic foraging cannot control for the amount of space required for production, and losses in the natural space available to forage have driven community transition to more controlled cultivation processes. When reliant on foraging, communities required much understanding about the environmental conditions that support mushroom growth and fruiting. Such traditional knowledge about environmental and agricultural inputs, whether explicit or tacit, has been adapted by communities for use in higher yield production systems that are less dependent of large natural spaces for production. The first steps towards the development of commercial production systems for a range of different wild saprophytic mushrooms in Uganda have already been taken, using traditional knowledge. The degree of control imparted by these production systems over environmental and agricultural inputs (as shown in Table 2), describes the current state of development of production systems for five wild saprophytic mushroom species. Environmental and agricultural inputs that have not been sufficiently controlled by existing production systems using traditional knowledge represent focus areas for future industry development.

Based on the data presented in Table 3, the most advanced area of development using traditional knowledge has been the identification of substrates for the successful cultivation of each of the mushroom species. This indicates that knowledge regarding nutrition requirements for mushroom growth and fruiting is well understood. For the production of *obunegyere* mushrooms in grassland agricultural areas, there is advanced indigenous knowledge of the association of termites with the substrate for growing *obunegyere* mushrooms. However, there is misunderstanding regarding the specific role that termites play in this production system. While it may be possible to develop a commercially viable substrate for this mushroom species without reliance on termites, this may present a significant challenge for industry development of *obunegyere* mushrooms.

Most importantly, for all artisanal mushroom production systems studied, communities have identified substrates that can be sourced at a sufficiently low price within each of their local regions to support the development of economically viable production systems in low or very low–income communities. This is an important finding given that substrates that are typically used in western production systems for commercial mushroom varieties are not necessarily available or cost effective within Sub-Saharan African communities, precluding their implementation and requiring identification of available cost-effective local analogues.

Also clear from existing artisanal production systems is that spawning method remains uncontrolled for each of the five wild mushroom species. Consequently, developing cost-effective spawning methods for each of the species is an essential next step for industry development, one that has been successfully achieved for community-based artisanal production of commercial oyster mushrooms. Oyster mushrooms have the most well-developed production system of all those studied. This is because oyster mushroom production has previously been a target of government-sponsored programs to develop artisanal production of mushrooms. The primary distinction between current artisanal production system for oyster mushrooms and the other listed wild mushrooms, is their cultivation within protected structures like enclosed spaces underneath houses. This cultivation method permits the control of environmental factors like, light, humidity, temperature, and, to some extent, control of contamination. The level of control achieved for the artisanal production of oyster mushrooms, through the use of simple building structures, indicates the economic feasibility of using covered structures to improve the degree of control over environmental and agricultural inputs for each of the production systems of the five studied wild mushroom species.

### Knowledge, practices, and beliefs

Artisanal mushroom production systems were analyzed with reference to the knowledge, practices, and beliefs that have supported their development in Kabale, Kamuli, Arua, Buikwe, and Mubende Districts of Uganda. The knowledge, practices, and beliefs that surround each of the production systems have environmental and cultural relevance beyond the economic relevance of income generation. Food security and healthcare are principle drivers for the maintenance of artisanal production systems, there being strong recognition within communities of the nutritional and medicinal value of mushrooms. Recognition of the environmental importance of reduced dependence on foraging is also strong within communities. There is clear acknowledgement that traditional foraging methods must change in response to decreasing availability of natural spaces. Furthermore, the advantages of overcoming the existing seasonality of artisanal mushroom production methods are well understood. Overcoming existing seasonal limitations would greatly help to drive the adoption of industry development activities directed at maintaining year-round mushroom production.

The strong role that women have in mushroom production is another key factor that would drive the development of gender equity through industry development of mushroom production. Women are integral within artisanal mushroom production systems, from owning the spaces needed for mushroom cultivation to having access to substrates for mushroom production and undertaking associated agroindustrial work such as *muramba* and tonto production. Women and landless marginalized groups like children can all benefit from mushroom production since it requires mainly indoor labor.

### Economic potential for mushroom production

Mushroom consumption has increased in recent years in Uganda and in other Sub-Saharan African countries, increasing consumer demand for mushrooms and intensifying the need for increased production capacity through improved mycoculture systems. In 1990, Ministry of Agriculture, Animal Industry and Fisheries of Uganda, introduced oyster mushroom production in the region. Mushroom production was targeted because it does not require ownership of large areas of land and the production cycle is short. Commercial mushroom production methods can protect against climate risks and seasonal variability. Furthermore, high mushroom prices can improve household income, alleviating poverty. Oyster mushroom spawn is supplied locally, with involvement from a University and a national agricultural research agency. The spawn is offered on a small scale on credit to artisanal producers in return for fresh mushrooms upon harvest. Over 10,000 farmers have been introduced to artisanal mushroom production in Kabale district alone [70]. Over 1600 mushroom farmers from the districts of Kabale, Kisoro, and Kanungu have participated in the mushroom spawn credit scheme. The participants of this study observed that unlike other cash crops, mushroom production is the most affordable because it requires less space and manpower, but it provides good income. Moreover, it is possible to develop value-added products to increase income and further improve the livelihoods of producers.

## Conclusions

Solely depending on existing production methods for increasing mushroom production is untenable in view of prevailing and increasing social, economic, and environmental pressures. However, much traditional knowledge, especially regarding substrate use, exists to improve the efficiency and capacity of the mycoculture of previously wild growing mushrooms. In addition to economic development, such integration of traditional knowledge into a mushroom industry development program would improve resource utilization and enhance conservation. When economic development is rooted in the existing social, economic, and environmental contexts of target communities, there is a multiplier effect across social, economic, and environmental domains. Each production system demonstrates the potential for commercial development. However, for each mushroom species, improved methods to control for environmental and agricultural inputs are necessary. A critical outcome of such improved control methods would be decoupling the production of mushrooms from seasonal limitations. Future industry development programs can now extend on existing knowledge of the required substrates for mushroom production. We recommend that future research and industry development programs should focus on methods for spore production, and improve growing methods using enclosed facilities or mycoculture-agroforestry as described for emponzira where felled logs can be spawned with spores and placed in designated spaces to enhance their growth. In doing so, the artisanal production of high value wild mushroom species can be developed in the same way that oyster mushrooms have even in the backyard.

Integrated growing of currently wild mushrooms by recycling the byproducts of other agricultural systems as substrates, improves livelihoods, provides nutritious products, and recycles biomass thus enhancing environment conservation. This study posits that new contexts for cultivation of wild mushroom species should be rooted in existing traditional innovation frameworks and organic ecosystems which mimic the natural ecosystems, and that multifaceted technological and organizational innovations are of value for subsistence agriculture intensification, agroecology conservation, and agro-industry development activity. Through sale of mushrooms and value-added mushroom products, there can be greater opportunities for low- and very low–income communities to participate in local economic market activity, and, in future, access international markets through the export of value-added products.

## Data Availability

The datasets generated and analyzed during the current study are not publicly available to preserve the anonymity of the participants in this study. Data are available from the corresponding author on reasonable request and with appropriate consideration of requirements for privacy.
